# Distribution study of *Chlamydia trachomatis *genotypes in symptomatic patients in Buenos Aires, Argentina: association between genotype E and neonatal conjunctivitis

**DOI:** 10.1186/1756-0500-3-34

**Published:** 2010-02-09

**Authors:** Lucía Gallo Vaulet, Carolina Entrocassi, Ana I Corominas, Marcelo Rodríguez Fermepin

**Affiliations:** 1 Inmunología Clínica, Departamento de Bioquímica Clínica, Facultad de Farmacia y Bioquímica, Universidad de Buenos Aires, Argentina; 2 Unidad de Estudios de Chlamydia y otras Infecciones del Tracto Genital, INFIBIOC - Instituto de Fisiopatología y Bioquímica Clínica, Universidad de Buenos Aires, Argentina; 3 Cátedra de Microbiología, Facultad de Farmacia y Bioquímica, Universidad de Buenos Aires, Argentina

## Abstract

**Background:**

*Chlamydia trachomatis *infections are the most prevalent sexually transmitted bacterial infections in the world. There is scarce data available referring to the distribution of *C. trachomatis *genotypes in Argentina. The aim of this study was to identify the genotypes of *C. trachomatis *circulating in the metropolitan area of Buenos Aires (Argentina) associated with *ophthalmia neonatorum *and genital infections.

**Findings:**

From 2001 to 2006, 199 positive samples for *C. trachomatis *infection from symptomatic adult patients and neonates with *ophthalmia neonatorum *from two public hospitals were studied. *C. trachomatis *genotypes were determined by PCR-RFLP of an ompA fragment.

Genotype E was the most prevalent regardless of the sample origin (46.3% 57/123 in adults and 72.4% 55/76 in neonates), followed by genotype D (19.5% 24/123) and F (14.6% 18/123) in adults, and G (9.2% 7/76) and D (7.9% 6/76) in neonates. We detected a significantly higher frequency of genotype E (p < 0.001, OR = 3.03 (1.57<OR<5.90)) in *ophthalmia neonatorum *than in genital specimens. Genotype D was associated with genital localization (p < 0.05, OR = 2.83 (1.03<OR<8.18)).

**Conclusion:**

We found a particularly increased frequency of *C. trachomatis *genotype E in neonatal conjunctivitis, which may indicate an epidemiological association between this genotype and the newborn population. The present study also contributed to increase the knowledge on genotype distribution of *Chlamydia trachomatis *in symptomatic adult patients in Buenos Aires, Argentina, in which genotypes E, D and F were the predominant ones.

## Introduction

*Chlamydia trachomatis *infections are the most prevalent sexually transmitted bacterial infections in the world. The World Health Organization estimated that 90 million new cases of chlamydial infections occurred globally in 1999 [1].

The prevalence of *C. trachomatis *lower tract infections varies from 2-25% according to the studied population, and is highest among adolescents [2]. Chlamydial infections are often asymptomatic (70-80% of women and up to 50% of men) and may therefore remain untreated. In women, an untreated infection can lead to pelvic inflammatory disease (PID), chronic pelvic pain, and, at a later stage, to ectopic pregnancy and tubal factor infertility [3].

Infants exposed to *C. trachomatis *at birth are at increased risk of developing eye and lung infections such as conjunctivitis and pneumonia and the estimated incidence of such infections in those newborn babies is 15% and 7% respectively [4-6]. Prevalence of neonatal conjunctivitis due to *C. trachomatis *has been reported to be about 8%, but it varies widely depending on prevalence values in their mothers [7-9].

*C. trachomatis *serotyping is based on immunogenic epitope analysis of the major outer membrane protein (MOMP), and it differentiates 18 serovars. Among these, serovars A to C are associated with trachoma, serovars D to K are common urogenital and ocular pathogens in adults and are also associated with chlamydial neonatal conjunctivitis worldwide, and serovars L1 to L3 are associated with lymphogranuloma venereum. Genotyping techniques are based on the analysis of the sequence changes in the single copy gene *omp*A that encodes MOMP. Genotype classification correlates with the serovar classification previously mentioned [10], but even though this classification is practical and accepted among researchers, does not allow establishing relationships between isolates. The application of new typing schemes based on sequencing a number of *C. trachomatis *variable genes, like MLST (Multi Locus Sequence Typing), will help to have a better understanding on the epidemiology and transmission of *C. trachomatis *infections [11].

*C. trachomatis *infection has not been largely studied in neonates regarding *ophthalmia neonatorum *and pneumonia [12-15] and data available on *C. trachomatis *serovar distribution associated to neonatal conjunctivitis is scarce. Datta et al. [16] studied *C. trachomatis *serovars in *ophthalmia neonatorum *in a trachoma endemic area, and reported that none of the identified serovars from infants with neonatal conjunctivitis belonged to the classic trachoma serovars. We have recently reported that serovar E was the most frequently detected serovar (71.0%) in a small population of neonates with neonatal conjunctivitis in Buenos Aires, Argentina [17].

The biological basis of the association between a cluster or a defined genotype of *C. trachomatis *and a particular disease is not yet well understood [18,19] and might be the result of many factors.

The aim of this study was to investigate the distribution of *C. trachomatis *genotypes among a population of patients showing symptoms of *ophthalmia neonatorum *and genital disease in the metropolitan area of Buenos Aires (Argentina).

## Methods

### Study Population

Samples included in this study were obtained from patients demanding diagnosis at the university hospital (Hospital de Clínicas "José de San Martín", University of Buenos Aires hospital, HJSM), located in downtown Buenos Aires, and at the national hospital (Hospital Nacional Prof Dr. A. Posadas, HNAP) located in the suburbs of Buenos Aires. Positive samples for *C. trachomatis *infection collected between January 2001 and December 2006 both from newborn babies (aged under 30 days) with *ophthalmia neonatorum *and from adults with genital symptoms were studied.

Patients were managed under standard approved hospital procedures. The population attending both hospitals belonged to lower and lower-middle classes and attended general practitioner's office. Neither STD clinic patients nor mother-child pairs were included in this study.

All neonates presented clinical signs of conjunctivitis. The most frequent symptoms described by adult patients from both hospitals included urethral or vaginal discharge, dyspareunia, dysuria, lower abdominal pain, and genital burning or itching sensation.

### Sample collection and detection of *C. trachomatis*

Ocular specimens from neonates and endocervical and urethral specimens from adult patients were collected using sterile Dacron tipped swabs.

All the samples obtained at the university hospital were placed in 2-sucrose phosphate (2SP) solution and were then analysed by *omp*A PCR and cultured in LLC-MK2 cells.

Specimens collected at the HNAP were analysed with two commercial ELISA tests. Between January 2001 and December 2004 Chlamydiazyme (Abbott Laboratories, Chicago IL) was used, and miniVIDAS (bioMerieux, Marcy l' Etoile, France) was used between January 2005 and December 2006. Antigen detection was performed according to the manufacturer's instructions. Fractions of ELISA-positive samples obtained in the period from 2001 to 2004 and from all samples obtained in 2005 and 2006 were frozen at -20°C and then transported in dry ice from the national hospital to the HJSM for further analysis by *omp*A PCR.

### Cell culture

Confluent cultures of LLC-MK2 cells (kindly provided by Sezione di Microbiologia DMCSS, Universitá degli Studi di Bologna, Bologna, Italy), grown at 37°C and 5% CO2 in culture media (minimum essential medium (MEM, Gibco), supplemented with 10% foetal calf serum (FCS, PAA Lab. GmbH, Austria), 0.1 mM non-essential amino acids (Gibco), 50 mg/L gentamicin, and 2 mM glutamine) were inoculated in duplicate with 500 μl of the 2SP sample suspension, and centrifuged at 700 g for 1 hour at 30°C. After a 72-hour incubation, one of each duplicate was methanol-fixed and stained with a genus-specific fluorescein-conjugated monoclonal antibody (Merifluor Chlamydia, Meridian Diagnostics Inc. Cincinnati, Ohio), following the manufacturer's instructions [20].

### OmpA PCR

The *omp*A gene of *C. trachomatis *was amplified using the methodology describe by Lan et al. [21]. Briefly, an approximately 1 kb fragment of the *omp*A gene was amplified using primers SERO1A (5'-ATG AAA AAA CTC TTG AAA TCG G-3') and SERO2A, (5'-TTT CTA GAT CTT CAT TCT TGT T-3'). The reaction was performed in a final volume of 50 μl containing 1.5 mM MgCl_2_, 0.05 mM of each deoxynucleotide triphosphate, 0.32 μM of each primer, 2 U of Taq DNA polymerase (Invitrogen Corporation, Brazil), and 10 μl of clinical specimen.

Cycling conditions began with an initial 7 min denaturation step at 94°C, followed by 40 cycles of denaturation at 95°C for 1 min, annealing at 45°C for 3 min, and extension at 72°C for 3 min. An additional 7-min extension at 72°C was performed at the end of the 40 cycles.

DNA of *C. trachomatis *L2/BU/434, (kindly provided by Sezione di Microbiologia DMCSS, Università degli Studi di Bologna, Bologna, Italy) and mock-infected cells were included as positive and negative controls respectively.

Then, 1 μl of the first-round PCR product was used for the semi-nested PCR, which was performed with the same reagents and conditions except for the primers, which were SERO2A, and one nested primer: PCTM3, (PCTM3: 5'-TCC TTG CAA GCT CTG CCT GTG GGG AAT CCT-3'). The PCR products of the second round were checked for correctness on ethidium-bromide stained 1.5% agarose gels. Positive results were routinely subjected to RFLP analysis, to determine the genotype and verify the absence of contamination with positive controls or cross-sample carry-over.

This semi-nested PCR was able to detect 1 to 10 inclusion-forming units in our laboratory conditions.

### RFLP genotyping

RFLP analysis of PCR-positive samples was carried out as described by Sayada et al. [22], using the semi-nested PCR product of approximately 1-Kbp. Briefly, 10 μl of the nested PCR product was digested with 2.5 U of *Alu*I (Promega, Madison, WI). Depending on the *Alu*I pattern, amplified samples were analyzed, if necessary, with *Hinf*I, *Dde*I or *Eco*RI (Promega, Madison, WI), according to the manufacturer's instructions.

Genotypes were identified by their restriction patterns on ethidium bromide stained 12% polyacrylamide gel electrophoresis.

Genotypes identified by RFLP from samples obtained between January 2005 and December 2006 were sent to *omp*A sequencing for verification, using the procedure of Jonsdottir et al [23].

### Statistical analysis

Epi Info version 2000 software (Center for Disease Control and Prevention, Atlanta, GA) was used to assess differences by application of the chi-square and exact binomial methods. Values of p ≤ 0.05 were considered statistically significant.

## Results

A total of 199 positive samples for *C. trachomatis *were collected from newborn babies and symptomatic adult patients attending the HJSM and HNAP between 2001 and 2006 (76 conjunctival sawbs, 42 male urethral scrapes and 81 cervical scrapes).

Over the 76 *omp*A positive samples detected among neonates, the genotypes identified and their distribution order were as follows: E (72.4%), G (9.2%), D (7.9%), F (5.3%), K (2.6%), H (1.3%) and I (1.3%).

Among the 81 positive endocervical swabs, the following genotypes were identified: E (46.9%), D (21.0%), F (16.1%), I (4.9%), K (4.9%), G (2.5%), Ja (2.5%) and H (1.2%). There was one mixed infection detected in an endocervical sample containing genotypes E and F.

The genotype distribution among male urethral samples was: E (45.2%), D (16.8%), F (11.9%), G (9.5%), I (9.5%), and K (7.1%).

**Figure 1:**
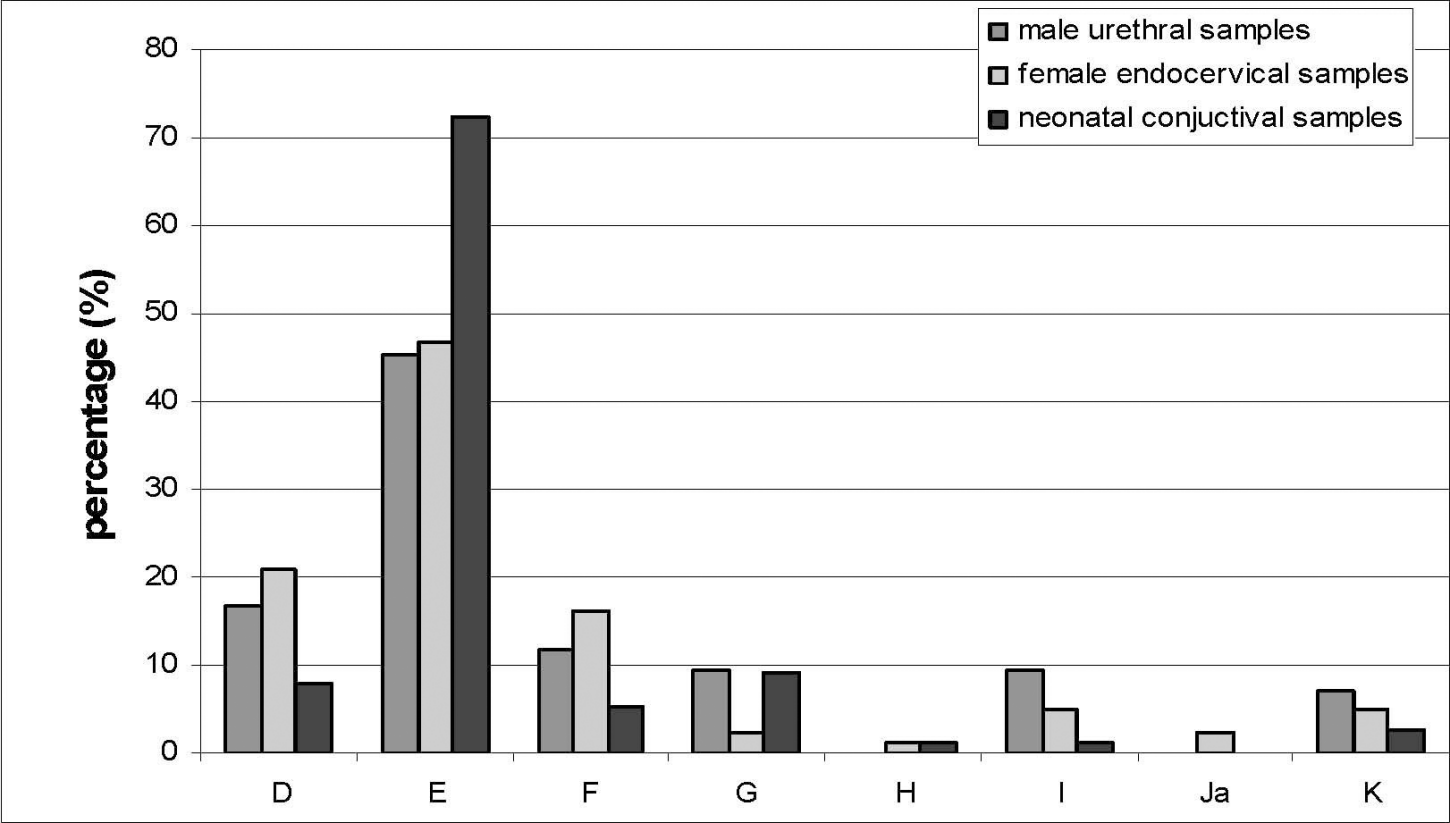
**Genotype distribution among 42 male urogenital samples, 81 female urogenital samples and 76 neonatal conjunctival samples**.

Neither genotypes A, B or C, nor L1 to L3 were detected in this study. The genotype distribution found in the two categories of examined samples, i.e. genital and ocular is given in Figure 1.

No significant differences on genotype distribution were found, neither between the two temporary subgroups from HNAP nor between the two hospitals. There were no significant differences between genders among genital genotypes (data not shown).

Altogether, the most frequently detected genotype was E, regardless of the sample's origin, but its frequency turned out to be significantly higher in neonatal ocular samples (p = 0.00056, OR = 3.03 (1.57<OR<5.90)) than in genital samples. Genotype D was negatively associated with ocular localization (p < 0.05, OR = 0.35 (0.11<OR<0.95)). The association of genotypes with ocular or genital disease are summarized respectively in Table 1.

**Table 1:** *C. trachomatis *genotype distribution according to sample origin.

	Type of sample		
		
Genotype detected	Neonatal conjunctival^a^ *n *(%)	Adult urogenital^b^ *n *(%)	*P *value
B Complex			
**D**	**6 (7.9)**	**24 (19.5)**	**0.043**
**E**	**55 (72.4)**	**57 (46.3)**	**0.00056**
Intermediate Group			
F	4 (5.3)	18 (14.6)	0.069
G	7 (9.2)	6 (4.9)	0.36
C Complex			
H	1 (1.3)	1 (0.8)	0.69
I	1 (1.3)	8 (6.5)	0.17
Ja	0	2 (1.6)	0.53
K	2 (2.6)	7 (5.7)	0.51

^a^76 neonatal conjunctival swabs.

^b^42 male urethral swabs, and 81 endocervical swabs. The distribution by gender is given in Figure 1.

## Discussion

In the present study we investigated the distribution of genotypes of *C. trachomatis*, which were obtained from newborn babies with *ophthalmia neonatorum *and symptomatic adult patients in a six-year period in two hospitals in Buenos Aires, Argentina.

Among symptomatic adults, genotypes E (45.2% in men and 46.9% in women), D (16.7% and 21.0%, respectively), and F (11.9% and 16.1%, respectively) were the most common ones, while H and Ja were rare. We found one mixed infection (0.5%) during this study, and this suggests a low prevalence of mixed infections in our population which is in agreement with the findings of Jonsdottir et al. [23], who detected 1% of mixed infections. The genotype distribution that we report in symptomatic adult patients was similar to previously reported ones [23,24], but different from that reported by other researchers where they found that genotype E was the most prevalent followed by F and D [25,26]. A limitation of the present study is that most of the samples were typed by RFLP and not *omp*A sequencing, so no distinction between D, Da and D variants was done. Although genotype D in our study is more prevalent than F, this could be the result from the fact that D also involves Da and D variant strains.

The high percentage of genotype E (72.4%) among neonatal ocular samples is in agreement with our previous report, where we found a predominance of this genotype in samples from newborn babies with neonatal conjunctivitis [17]. Even though these findings could be due to the genital predominance of genotype E, this fact might not be the only explanation of the higher detection rate of this genotype in neonatal conjunctivitis.

In principle, our findings could be biased by the utilization of an ELISA assay as the only diagnostic test used at the HNAP from 2001 to 2004, which could have resulted in false negative findings in a substantial portion of the samples studied. However, even when all samples from the HJSM were studied both by culture and a sensitive PCR, we did not find significant differences on the genotype distribution among adults between both hospitals. Moreover, all samples from the HNAP collected from 2005 to 2006 were tested by ELISA and *omp*A PCR, and we did not find significant differences in genotype distribution compared with the one obtained for the 2001-2004 period. Hence, Gomes et al. [27] have shown that there is no association between genotype and chlamydial load at the infection site in genital specimens, which also supports the notion that the sensitivity of ELISA did not biased the genotype distribution. Another possible limitation of this study is that we only studied adult symptomatic patients and newborn babies with clinical signs of conjunctivitis, which could have biased our results. However other study findings in asymptomatic population have found frequencies of genotype E similar to ours [25].

We hypothesize that some kind of selective pressure could explain the high frequency of genotype E in neonatal conjunctivitis. On the one hand, it may be reflecting the difference in the immune status between neonates and adults. This is supported by the fact that there is a difference in the distribution of genital genotypes correlated with the patients' age and with a predominance of genotype E in adolescents [26], whereas there is an equal correlation in the distribution of *C. trachomatis *genotypes between urogenital and adult conjunctivitis eye samples [28,29]. On the other hand, *C. trachomatis *genotype E could have an enhanced capability of infecting the conjunctival mucosa of neonates as compared to other genotypes or a differential mother-child transmission dynamic over other genotypes. This idea is supported by the negative association of genotype D with *ophthalmia neonatorum*, given that this serovar is the second most frequent one in our adult female population but the third one in neonatal ocular samples. In order to confirm this negative association, the distinction between D, Da and D variants would be necessary.

It is possible that the higher frequency of genotype E could be due to distinctive characteristic of some strains of this genotype. The analysis of portions of the *C. trachomatis *genome which are rich in SNPs (single nucleotide polymorphisms) [30], as well as the application of new typing schemes based on sequencing a number of *C. trachomatis *variable genes (MLST) [11] may provide a better understanding of this phenomenon. Furthermore, an assessment of specific immunity in different age groups and in vitro studies of infectivity and citotoxicity of different genotypes in conjunctival cell lines may also help to clarify our hypothesis.

## Conclusions

This study confirmed an increased frequency of genotype E in neonatal ocular samples. The present study also contributed to increase the knowledge on genotype distribution of *C. trachomatis *in adult symptomatic patients that attended two public hospitals in Buenos Aires city, Argentina, in which genotypes E, D and F were the predominant ones. This genotype distribution constitutes one of the few reports of this kind in Latin America.

## Competing interests

The authors declare that they have no competing interests.

## Authors' contributions

MLGV and ACE have equally contributed to the developing of this study. MRF designed the study and MLGV and AIC drafted the manuscript. MRF reviewed the manuscript. All authors have read and approved of the final manuscript.
